# Identifying Younger Postmenopausal Women With Osteoporosis Using USPSTF-Recommended Osteoporosis Risk Assessment Tools

**DOI:** 10.1001/jamanetworkopen.2025.0626

**Published:** 2025-03-18

**Authors:** Henry W. Zheng, Alex A. T. Bui, Kristine E. Ensrud, Nicole C. Wright, JoAnn E. Manson, Nelson B. Watts, Karen C. Johnson, Aladdin H. Shadyab, Carolyn J. Crandall

**Affiliations:** 1 Medical and Imaging Informatics, UCLA (University of California, Los Angeles), Los Angeles; 2 Departments of Radiological Sciences, Bioengineering and Bioinformatics, David Geffen School of Medicine at UCLA, Los Angeles; 3 Division of Epidemiology and Community Health, Department of Medicine, University of Minnesota, Minneapolis; 4 Department of Epidemiology, School of Public Health, University of Alabama at Birmingham; 5 Department of Medicine, Brigham and Women’s Hospital, Harvard Medical School, Boston, Massachusetts; 6 Bone Health and Osteoporosis Services, Bon Secours Mercy Health, Cincinnati, Ohio; 7 Department of Preventive Medicine, University of Tennessee Health Science Center, Memphis; 8 Herbert Wertheim School of Public Health and Human Longevity Science and Division of Geriatrics, Gerontology, and Palliative Care, Department of Medicine, University of California, San Diego, La Jolla; 9 Department of Medicine, David Geffen School of Medicine at UCLA, Los Angeles

## Abstract

**Importance:**

For younger postmenopausal women, clinical guidelines recommend using osteoporosis risk prediction tools to identify candidates with low bone mineral density (BMD). However, the performance of these tools is not well quantified.

**Objective:**

To examine the performance of Osteoporosis Risk Assessment Instrument (ORAI) and Osteoporosis Index of Risk (OSIRIS), compared with Osteoporosis Self-Assessment Tool (OST), in identifying the presence of osteoporotic BMD in younger postmenopausal women.

**Design, Setting, and Participants:**

This cross-sectional study used data from the Women’s Health Initiative Bone Density Substudy, which was conducted at 3 clinical centers in Tucson and Phoenix, Arizona; Pittsburgh, Pennsylvania; and Birmingham, Alabama. Participants were healthy postmenopausal women aged 50 to 64 years with BMD measurements evaluated using the 3 risk prediction tools: OSIRIS, ORAI, and OST. Risk factors and other participant characteristics were compared across osteoporosis status. Data were collected from October 1993 to December 1998 and analyzed between September 23, 2023, and April 10, 2024.

**Exposures:**

The primary exposures were OSIRIS, ORAI, and OST risk scores.

**Main Outcomes and Measures:**

Primary outcome was osteoporosis defined by BMD T score of –2.5 or lower at 1 or more of 3 anatomical locations: femoral neck, total hip, and/or lumbar spine. The tools were evaluated via area under the receiver operating characteristic curve (AUC) at published score cutoffs and at alternate cutoffs.

**Results:**

Among 6067 included participants (mean [SD] age at baseline, 57.7 [4.1] years), the prevalence of osteoporosis was 14.1% (n = 857) at any 1 of 3 anatomical sites. AUC for identifying osteoporosis at any site was 0.633 (95% CI, 0.633-0.634) for OSIRIS, 0.663 (95% CI, 0.663-0.664) for ORAI, and 0.654 (95% CI, 0.654-0.655) for OST.

**Conclusions and Relevance:**

In this cross-sectional study, 3 guideline-recommended osteoporosis risk assessment tools had fair to moderate discrimination in identifying osteoporosis defined by lowest BMD at any 1 of 3 skeletal sites. Screening is essential to reducing individual and societal burden of osteoporosis and related fractures, and this study showed a gap in identifying younger postmenopausal women using common clinical risk factors.

## Key Points

**Question:**

How well do osteoporosis risk assessment tools recommended by the US Preventive Services Task Force perform in identifying osteoporotic bone mineral density (BMD) in younger postmenopausal women?

**Findings:**

In this cross-sectional study of 6067 healthy postmenopausal women aged 50 to 64 years, 3 tools (Osteoporosis Index of Risk, Osteoporosis Risk Assessment Instrument, and Osteoporosis Self-Assessment Tool) had fair to moderate discrimination in identifying osteoporosis as defined by lowest BMD at any 1 of 3 skeletal sites.

**Meaning:**

Findings of this study suggest that the performance of these 3 tools was suboptimal in identifying osteoporosis defined by lowest BMD at the hip or lumbar spine in younger postmenopausal women.

## Teaser

This cross-sectional study examines the performance of 3 osteoporosis screening tools recommended by the US Preventive Services Task Force (USPSTF)—the Osteoporosis Risk Assessment Instrument, the Osteoporosis Index of Risk, and the Osteoporosis Self-Assessment Tool[em dash] among US women aged 50 to 64 years.

## Introduction

Osteoporosis is responsible for 3 million fractures per year in the US,^1^ exceeding the annual number of new cases of myocardial infarction (805 000), breast cancer (310 720), and prostate cancer (299 010) combined.^2,3,4^ Annual direct costs of osteoporosis in the US are $17 billion per year,^5^ with projections of over $95 billion by 2040.^6^ As osteoporosis risk increases with advancing age, and because there is effective drug therapy to reduce fracture risk in postmenopausal women with osteoporosis (defined by a bone mineral density [BMD] T score of −2.5 or lower), both the Bone Health and Osteoporosis Foundation and US Preventive Services Task Force (USPSTF) recommend routine screening for osteoporosis in women aged 65 years or older.^1,7^ The USPSTF recommends the use of a clinical risk assessment tool to identify candidates for screening in women younger than 65 years.^7^ The 5 clinical risk assessment tools recommended by the USPSTF are the Osteoporosis Risk Assessment Instrument (ORAI),^8,9,10^ the Osteoporosis Index of Risk (OSIRIS),^11,12^ the Osteoporosis Self-Assessment Tool (OST),^8,10,13,14^ the Fracture Risk Assessment Tool (FRAX),^15^ and the Simple Calculated Osteoporosis Risk Estimation Tool (SCORE).^16^ Previous studies have examined the performance of 3 of the 5 recommended tools (FRAX,^17,18,19,20,21,22^ OST,^17,21,22^ and SCORE^21,22^) in identifying BMD T score of –2.5 or lower and/or subsequent fracture risk among postmenopausal women younger than 65 years. However, OSIRIS and ORAI have not been examined.

Previous national and international studies on ORAI focused on fracture risk discrimination in older women and/or involved participants aged 60 years or older,^8,9,10,13,23,24,25,26^ whether or not they included participants residing in the US.^10,13,24^ The studies evaluating the discrimination of OSIRIS have been conducted in international populations and showed area under the receiver operating characteristic curve (AUC) values between 0.71 and 0.73.^11,26^ Little information is available regarding the performances of ORAI and OSIRIS in identifying osteoporosis based on BMD in postmenopausal women aged 50 to 64 years for whom USPSTF recommended the use of formal risk assessment tools for screening decisions.^7^ Therefore, the goal of this study was to examine the performance of ORAI and OSIRIS compared with OST in identifying the presence of osteoporotic BMD in younger postmenopausal women.

## Methods

### Data Source and Participants

We performed a cross-sectional study using data from the Women’s Health Initiative (WHI), a large-scale prospective study of postmenopausal women. Across 40 US clinical centers, healthy postmenopausal women aged 50 to 79 years were enrolled in either the WHI observational study or the WHI clinical trials and were followed up for over 30 years using a battery of health, lifestyle, and psychosocial metrics.^27^ The WHI clinical trial was composed of 3 randomized clinical trials studying low-fat dietary modification, menopausal hormone therapy, and calcium and vitamin D supplementation. The goal of the WHI observational study was to evaluate common causes of morbidity and mortality, particularly cardiovascular, cancer, and osteoporotic outcomes. The Institutional Review Board at each enrolling institution approved the WHI. All participants provided written informed consent. WHI approval and participant consent also apply to the present study. We followed the Strengthening the Reporting of Observational Studies in Epidemiology (STROBE) reporting guideline.

The WHI Bone Density Substudy measured osteoporosis at the total hip, femoral neck, and lumbar spine via BMD measurements at 3 of 40 clinical centers (Tucson and Phoenix, Arizona; Pittsburgh, Pennsylvania; and Birmingham, Alabama). Participants (n = 11 461 women) underwent hip and anteroposterior lumbar spine BMD testing via dual-energy x-ray absorptiometry (DXA; Hologic QDR 2000 or QDR 4500 [Hologic Inc]). Standard protocols were used for positioning and analysis of DXA measurements.^28^ Quality assurance included review of lumbar spine and hip phantom scans at each center, use of calibration phantoms across clinical sites, flagging of scans with specific problems, and review of a random sample of all scans.

The current study analyzed WHI Bone Density Substudy participants younger than 65 years who reported at baseline that they were not using prescription osteoporosis drug therapies (bisphosphonate, calcitonin, or selective estrogen receptor modulator) and who had available data for calculation of osteoporosis risk. Data were collected from October 1993 to December 1998^29^ and analyzed between September 23, 2023, and April 10, 2024.

### Outcomes

In line with routine clinical practice and osteoporosis treatment guidelines, we established the primary outcome of this study as osteoporosis defined by a BMD DXA measurement T score of –2.5 or lower at 1 or more of 3 anatomical locations: femoral neck, total hip, and lumbar spine.^1,30^ T scores were derived from the National Health and Nutrition Examination Survey III phase 1 normative reference database per the World Health Organization 2007 technical report.^31,32^ The secondary outcome was osteoporosis defined by a DXA measurement T score of –2.5 or lower only at the femoral neck.

### Clinical Risk Assessment Tools

The 3 clinical risk assessments tools examined in this study were OSIRIS, ORAI, and OST (formulas provided in the eTable in Supplement 1). OSIRIS scores are based on age, weight (kilogram), current estrogen use, and prior low-impact fracture.^11^ ORAI scores are based on age, weight (kilogram), and use of estrogen.^9^ OST scores are based on weight (kilogram) and age (0.2 × [body weight (kilogram) – age [years]), truncated to yield an integer.^8,13,14^ We calculated OSIRIS, ORAI, and OST scores using the current study cohort’s baseline data. Weight was measured at each clinical center using standardized protocols. Current use of estrogen (oral or transdermal) and history of fractures were reported via the baseline self-assessment questionnaire. The primary analysis examined guidelines-recommended score cutoffs or thresholds for the tools (OSIRIS score <1, ORAI score >8, and OST score <2) in predicting osteoporosis.^7,9,11,13^

### Other Participant Characteristics

On baseline self-assessment questionnaires, we collected data regarding race, ethnicity, body mass index (BMI), smoking status (never, past, or current), alcohol intake, number of falls in the past year (0, 1, 2, or ≥3), and physical function. Race was self-identified by participants and categorized as American Indian or Alaska Native, Asian and Native Hawaiian or Other Pacific Islander, Black, White, or multiracial. Ethnicity was self-reported as Hispanic or not Hispanic. Race and ethnicity were collected because of existing clinical evidence that they might be risk factors for osteoporosis.^17^ BMI was calculated as weight in kilograms divided by height in meters squared and included under 25, 25 to under 30, and 30 or higher. Participants’ self-reported alcohol intake was categorized as never (<12 alcoholic drinks in lifetime), past (>12 alcoholic drinks over lifetime but <1 alcoholic drink per month currently), and current (≥1 alcoholic drinks per month).

### Statistical Analysis

We ascertained the characteristics of the participants overall and by osteoporosis status. We used χ^2^ tests to compare participant characteristics by baseline osteoporosis status. We calculated sensitivity, specificity, positive predictive value (PPV), and discrimination via AUC for distinguishing participants with osteoporosis (T score of –2.5 or lower) from those without osteoporosis (T score higher than –2.5). We used logistic regression to calculate the AUC for OSIRIS, ORAI, and OST at their published score cutoffs (OSIRIS <1,^11^ ORAI >8,^9^ and OST <2^13^) for predicting BMD T score of –2.5 or lower, at alternative cutoffs calibrated to correspond to sensitivity of 80% or higher, and at the cutoff corresponding to the maximum AUC value. The AUC is a measure of discrimination. In the context of BMD T score of –2.5 or lower, discrimination quantifies the ability to differentiate between women with and women without a BMD T score of –2.5 or lower. An AUC value of 0.5 indicates no discrimination (ie, the tool is no better than chance in discriminative ability), values between 0.5 and 0.7 indicate poor to fair discrimination, values between 0.7 and 0.8 indicate acceptable discrimination, and values between 0.8 and 0.9 indicate good discrimination.^33^ CIs for performance metrics were calculated via 1000-resample bootstrapping.

Two-sided *P* < .05 indicated statistical significance. Statistical analysis was performed using Python 3.0 (Python Software Foundation).

## Results

**Figure 1.**
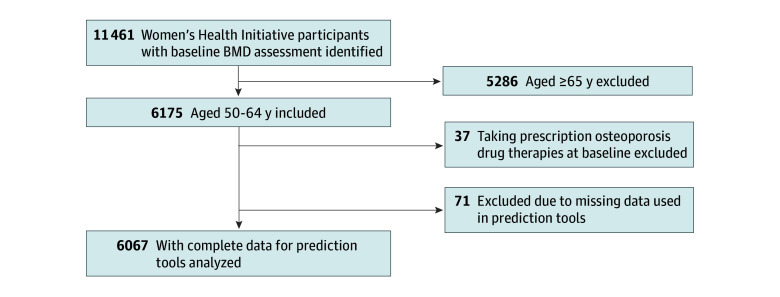
Analytic Flow Diagram BMD indicates bone mineral density.

Of the 11 461 women in the WHI Bone Density Substudy, 5286 were excluded for being 65 years or older, 37 for receiving osteoporosis drug therapies at baseline, and 71 for missing needed data (Figure 1). Thus, the final analytic sample consisted of 6067 participants with a mean (SD) age of 57.7 (4.1) years, of whom 86 (1.5%) self-identified as American Indian or Alaska Native, 30 (0.5%) as Asian and Native Hawaiian or Other Pacific Islander, 965 (16.7%) as Black, 529 (8.8%) as Hispanic, 4626 (80.2%) as White, 59 (1.0%) as multiracial, and 5473 (91.2%) as non-Hispanic individuals. A total of 2132 women (35.3%) had a BMI of 30 or higher (Table 1).

**Table 1.** Baseline Characteristics of Study Participants Overall and by Osteoporosis Status^a^

Characteristics	All participants, No. (%) (N = 6067)	Participants with BMD T score –2.5 or lower at 3 locations, No. (%) (n = 857)	Participants without T score higher than –2.5 at 3 locations, No. (%) (n = 5210)	*P* value^b^
Age, mean (SD), y	57.7 (4.1)	59.3 (3.7)	57.5 (4.2)	<.001
Race, self-reported				
American Indian or Alaska Native	86 (1.5)	10 (1.2)	76 (1.5)	.007
Asian and Native Hawaiian or Other Pacific Islander	30 (0.5)	7 (0.9)	23 (0.5)	
Black	965 (16.7)	174 (21.2)	791 (16.0)	
White	4626 (80.2)	623 (75.9)	4003 (81.0)	
More than 1 race	59 (1.0)	7 (0.9)	52 (1.1)	
Ethnicity, self-reported				
Hispanic	529 (8.8)	64 (7.5)	465 (9.0)	.18
Not Hispanic	5473 (91.2)	787 (92.5)	4686 (91.0)	
BMI				
<25	1894 (31.3)	437 (51.4)	1457 (28.1)	<.001
25 to <30	2019 (33.4)	272 (32.0)	1747 (33.6)	
≥30	2132 (35.3)	142 (16.7)	1990 (38.3)	
Weight, mean (SD), kg	76.06 (17.24)	67.25 (14.26)	77.52 (17.26)	<.001
Smoking status				
Never	3170 (52.8)	473 (55.6)	2697 (52.4)	.007
Past	2238 (37.3)	277 (32.5)	1961 (38.1)	
Current	592 (9.9)	101 (11.9)	491 (9.5)	
Alcohol intake^c^				
Never	967 (16.1)	171 (20.1)	796 (15.4)	<.001
Past	2150 (35.8)	205 (24.1)	1108 (21.5)	
Current: ≥1 drink/mo	2881 (48.0)	476 (55.9)	3259 (63.1)	
Estrogen and/or estrogen + progestogen therapy use currently at baseline (oral or transdermal)				
Yes	2641 (43.5)	226 (26.4)	2415 (46.4)	<.001
No	3426 (56.5)	631 (73.6)	2795 (53.6)	
No. of falls in the past year^d^				
0	3515 (66.2)	506 (67.9)	3009 (66.0)	.72
1	1103 (20.8)	152 (20.4)	951 (20.8)	
2	433 (8.2)	55 (7.4)	378 (8.3)	
≥3	256 (4.8)	32 (4.3)	224 (4.9)	
History of fracture at age ≥55 y^e^				
Yes	320 (5.3)	86 (10.0)	234 (4.5)	<.001
No	3122 (51.5)	473 (55.2)	2649 (50.8)	
NA (age <55 y)	2625 (43.3)	298 (34.8)	2327 (44.7)	

Abbreviations: BMD, bone mineral density; BMI, body mass index (calculated as weight in kilograms divided by height in meters squared); NA, not applicable.

^a^ Participants may be missing data on certain covariates, leading to lower total.

^b^ *P* value comparing characteristic by BMD T score of –2.5 or lower (yes/no) used *t* tests for continuous variables and χ^2^ tests for categorical variables.

^c^ One drink was based on a medium serving size of 12 oz of beer, 6 oz of wine, or 1.5 oz of liquor.

^d^ “During the past 12 months, how many times did you fall and land on the floor or ground?”

^e^ Locations of fractures considered were hip, thoracic or lumbar vertebra, humerus, lower arm and wrist, hand excluding fingers, lower leg or ankle, and foot excluding toes.

Baseline osteoporosis was present in 857 participants (14.1%) at any of 3 anatomical sites and in 300 participants (4.9%) at the femoral neck. Compared with women without osteoporosis, women with osteoporosis were more likely to be older, have lower weight (kilograms) and BMIs, be current smokers and drink alcohol, report a history of fracture and to be less likely to report estrogen use at baseline (Table 1). Prevalence of osteoporosis also varied by race and ethnicity.

### Evaluation of Risk Assessment Tools

Table 2 compared the performance of the 3 tools across all thresholds at any site vs at the femoral neck alone. All 3 tools were better able to predict a BMD T score of –2.5 or lower at the femoral neck than at any site. At any threshold, OSIRIS yielded an AUC of 0.830 (95% CI, 0.829-0.830), ORAI yielded an AUC of 0.805 (95% CI, 0.805-0.806), and OST yielded an AUC of 0.818 (95% CI, 0.817-0.819).

**Table 2.** Area Under the Receiver Operating Characteristic Curve Values for ORAI, OSIRIS, and OST Across All Score Cutoffs for Predicting Risk of BMD T Score of –2.5 or Lower

Outcome: BMD T score −2.5 or lower	Participants, No. (%)	Events, No. (%)	AUC (95% CI)		
			ORAI	OSIRIS	OST
Femoral neck	6067 (100)	300 (4.9)	0.805 (0.805-0.806)	0.830 (0.829-0.830)	0.818 (0.817-0.819)
Any BMD site^a^	6067 (100)	857 (14.1)	0.714 (0.713-0.715)	0.741 (0.740-0.741)	0.716 (0.715-0.716)

Abbreviations: AUC, area under the receiver operating characteristic curve; BMD, bone mineral density; ORAI, Osteoporosis Risk Assessment Instrument; OSIRIS, Osteoporosis Index of Risk; OST, Osteoporosis Self-Assessment Tool.

^a^ Femoral neck, total hip, or lumbar spine.

Using published score thresholds, OSIRIS yielded a sensitivity of 37.8% (95% CI, 37.8%-38.0%), specificity of 88.8% (95% CI, 88.7%-88.8%), PPV of 35.6% (95% CI, 35.5%-35.7%), and AUC of 0.633 (95% CI, 0.633-0.634). ORAI yielded a sensitivity of 53.3% (95% CI, 53.2%-53.4%), specificity of 79.4% (95% CI, 79.3%-79.4%), PPV of 29.8% (95% CI, 29.7%-29.9%), and AUC of 0.663 (95% CI, 0.663-0.664). OST yielded a sensitivity of 62.4% (95% CI, 62.3%-62.5%), specificity of 68.5% (95% CI, 68.4%-68.5%), PPV of 24.5% (95% CI, 24.5%-24.6%), and AUC of 0.654 (95% CI, 0.654-0.655) (Table 3). These were values for identifying women with osteoporosis, as defined by lowest BMD at any of the 3 measured locations.

**Table 3.** Estimating BMD T Score of –2.5 or Lower for Any BMD Site at Published and Alternative Score Cutoffs Calibrated to Sensitivities^a^

Assessment tool at score cutoff	Performance metric (95% CI), %			AUC (95% CI)
	Sensitivity	Specificity	PPV	
OSIRIS				
<1	37.8 (37.8-38.0)	88.8 (88.7-88.8)	35.6 (35.5-35.7)	0.633 (0.633-0.634)
<4	78.3 (78.2-78.4)	56.0 (56.0-56.0)	22.6 (22.6-22.7)	0.671 (0.671-0.672)
<6	90.9 (90.9-91.0)	33.1 (33.0-33.1)	18.2 (18.2-18.3)	0.620 (0.620-0.621)
<8	95.9 (95.9-96.0)	17.5 (17.5-17.5)	16.0 (16.0-16.0)	0.567 (0.567-0.567)
<13	99.1 (99.1-99.1)	2.8 (2.8-2.8)	14.3 (14.3-14.4)	0.509 (0.509-0.510)
ORAI				
>8	53.3 (53.2-53.4)	79.4 (79.3-79.4)	29.8 (29.7-29.9)	0.663 (0.663-0.664)
>6	81.7 (81.6-81.8)	46.3 (46.3-46.4)	20.0 (20.0-20.0)	0.640 (0.640-0.641)
>4	92.0 (92.0-92.1)	26.5 (26.5-26.6)	17.1 (17.0-17.1)	0.592 (0.592-0.593)
>2	93.9 (93.9-94.0)	21.5 (21.5-21.5)	16.4 (16.4-16.5)	0.577 (0.577-0.577)
>1	98.2 (98.2-98.3)	9.5 (9.5-9.5)	15.1 (15.1-15.2)	0.539 (0.539-0.539)
OST				
<2	62.4 (62.3-62.5)	68.5 (68.4-68.5)	24.5 (24.5-24.6)	0.654 (0.654-0.655)
<4	84.2 (84.1-84.3)	43.2 (43.2-43.3)	19.6 (19.5-19.6)	0.637 (0.637-0.637)
<5	89.1 (89.1-89.2)	33.2 (32.0-33.3)	18.0 (17.9-18.0)	0.612 (0.611-0.612)
<7	96.0 (96.0-96.1)	17.9 (17.9-17.9)	16.1 (16.1-16.1)	0.570 (0.569-0.570)
<13	99.1 (99.1-99.1)	2.0 (2.0-2.0)	14.2 (14.2-14.3)	0.505 (0.505-0.506)

Abbreviations: AUC, area under the receiver operating characteristic curve; BMD, bone mineral density; ORAI, Osteoporosis Risk Assessment Instrument; OSIRIS, Osteoporosis Index of Risk; OST, Osteoporosis Self-Assessment Tool; PPV, positive predictive value.

^a^ 95% CIs calculated via 1000-iteration resampled bootstrapping.

**Figure 2.**
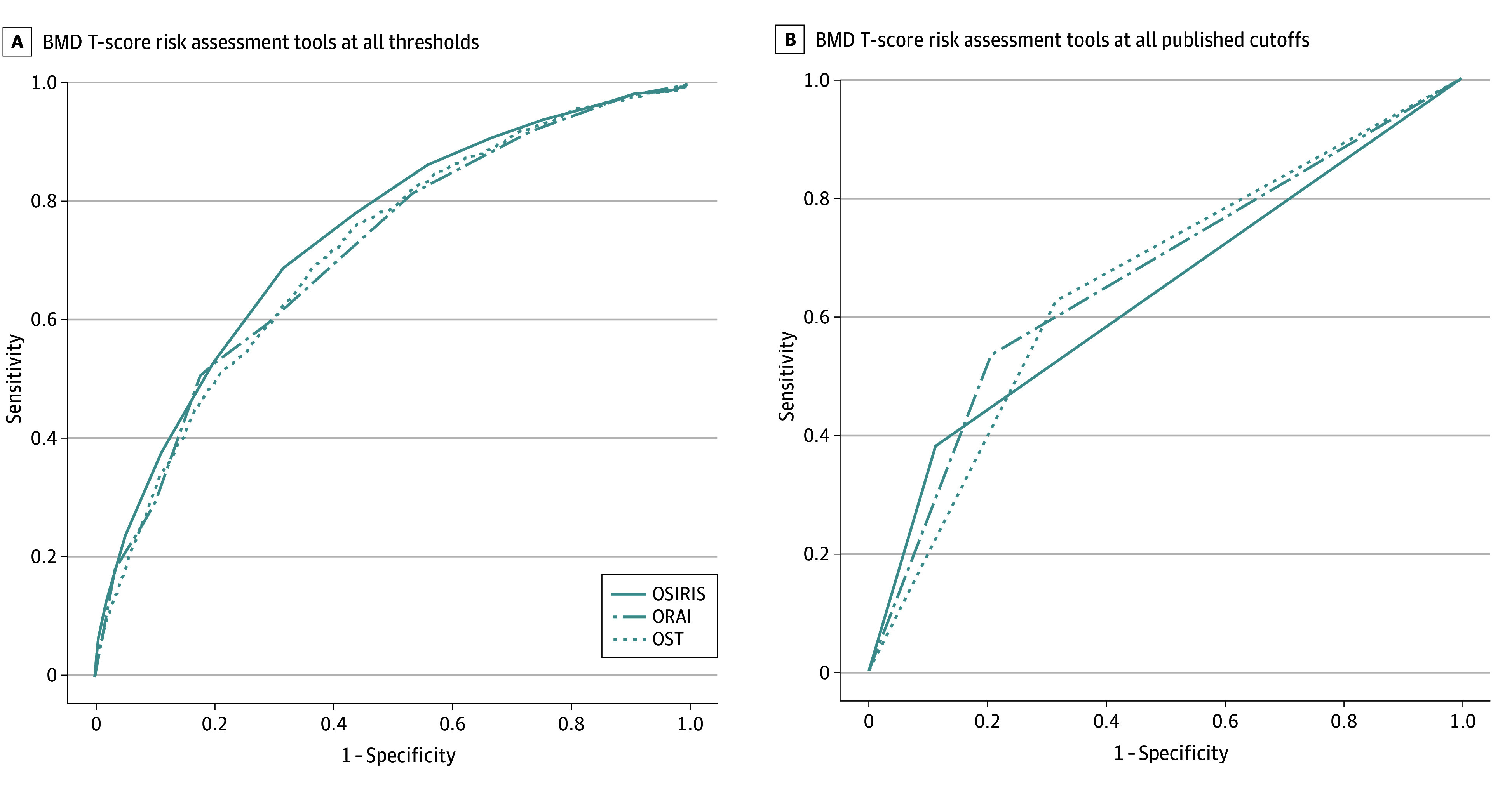
Receiver Operating Characteristic Curves of US Preventive Services Task Force–Recommended Osteoporosis Risk Assessment Tools in Women Aged 50 to 64 Years BMD indicates bone mineral density; ORAI, Osteoporosis Risk Assessment Instrument; OSIRIS, Osteoporosis Index of Risk; and OST, Osteoporosis Self-Assessment Tool.

Contextualized to 1000 theoretical postmenopausal women aged 50 to 64 years, 141 (14.1%) would have osteoporosis, of whom OSIRIS would identify 51 (based on 37.8% sensitivity on 141 positive cases), ORAI would identify 75 (based on 53.3% sensitivity), and OST would identify 87 (based on 62.4% sensitivity). Conversely, 859 women (85.9%) would not have osteoporosis, but OSIRIS would lead to 94 extraneous BMD tests, ORAI would lead to 180 extraneous tests, and OST would lead to 266 extraneous tests. There was no noticeable difference in discriminative ability among the 3 tools at all score thresholds (Figure 2A) or at their published dichotomized thresholds (Figure 2B) at any of the 3 locations, although performance was superior at any threshold. Table 3 characterized the performance of the prediction tools at published and alternative cutoffs calibrated to sensitivity of 80% or greater. AUC was optimized in the sample for the following score cuttoffs: OSIRIS at lower than 3, ORAI at higher than 9, and OST at lower than 3. However, even at these optimized thresholds, the maximum AUC for a given tool for predicting osteoporosis based on BMD T score of –2.5 or lower did not exceed 0.70 for at least 1 skeletal site, indicating only fair discrimination at best.

## Discussion

In this cross-sectional study, we compared the predictive utility of 3 guideline-recommended osteoporosis calculators—ORAI, OSIRIS, and OST—for identifying osteoporosis in postmenopausal women aged 50 to 64 years. Discrimination of ORAI or OSIRIS were less than ideal in identifying osteoporosis at the hip or the lumbar spine, with AUCs of 0.741 or lower across all score thresholds. At best, the published and alternative cutoffs evaluated yielded only fair to moderate discrimination between women with vs women without osteoporosis. By comparison, the AUC for OST was 0.716 at all thresholds or 0.653 at the guideline-recommended threshold, highlighting that adding risk factors in ORAI (estrogen use) and in OSIRIS (estrogen use and history of fracture) not included in OST did not appreciably increase overall discrimination, merely reconfiguring the tradeoff between sensitivity and specificity. In contrast, discrimination in identifying osteoporosis at the femoral neck was good for all 3 tools, with AUCs across all thresholds ranging from 0.818 to 0.830.

The study also showed equivalencies in performance among the 3 tools. The AUC for OSIRIS was marginally higher than that for ORAI and OST, but the difference was not large enough to be clinically meaningful. Meanwhile, OST’s simplicity in having only weight and age as risk factors is an advantage for practical use. While the performance of each tool in identifying osteoporosis at the femoral neck was acceptable, their less-than-ideal performance in identifying osteoporosis defined by lowest BMD at the lumbar spine, femoral neck, and/or total hip raises doubt on their capability to identify osteoporosis as typically defined in clinical practice in women aged 50 to 64 years.

While previous studies of these osteoporosis risk prediction tools are lacking in the US, the results of this study are in general agreement with findings of similar studies in other countries, such as an evaluation of OSIRIS, ORAI, and OST in 665 Spanish women aged 40 to 60 years and research in 207 British women reporting AUC values similar to those in the present study.^34,35^ Between these studies, different thresholds for OSIRIS and ORAI were evaluated that yielded tradeoffs in sensitivity to specificity without substantial improvements in AUC, while no differences in discrimination were observed distinguishing OSIRIS from ORAI or OST. Within the US, a study of 226 postmenopausal women aged 45 years or older reported a sensitivity of 0.68 (95% CI, 0.49-0.88) and an AUC of 0.74 (95% CI, 0.63-0.84) for ORAI at an over 8 score cutoff to predict BMD T score of –2.5 or lower at the lumbar spine and/or total hip.^24^ A second US study of 290 women aged 50 to 64 years recruited from a single institution reported an AUC of 0.60 for ORAI to identify a BMD T score of –2.5 or lower at the lumbar spine and/or femoral neck.^36^ Another study of 445 women aged 50 to 65 years recruited at a single institution achieved a higher sensitivity of 74% (95% CI, 57%-87%) and an AUC of 0.69 (95% CI, 0.60-0.78).^37^

While ORAI’s performance appears better in these smaller studies, these previous studies were conducted in more homogeneous populations, whereas the present study was conducted in a larger and more diverse population. Additionally, a US study of 202 women aged 45 to 64 years recruited from a single city reported an AUC of 0.82 under ORAI for identifying a BMD T score of –2.5 or lower at the femoral neck.^38^ For OSIRIS, the original publication did not include younger women and studies in US women have not involved postmenopausal women aged 65 years or younger. These tools were designed and calibrated for prediction of BMD at the femoral neck. However, risk for osteoporosis is usually identified in practice using measures at multiple skeletal sites, and this study found suboptimal performance from these tools in predicting osteoporosis risk when generalized to other BMD anatomical sites, highlighting the innovation of the current study.

A previous study compared 2 other guideline-recommended osteoporosis risk prediction tools—FRAX (designed for fracture prediction) and SCORE (designed for identifying osteoporosis by BMD)—against OST in WHI participants aged 50 to 64 years and reported AUCs of 0.60 (95% CI, 0.56-0.63), 0.72 (95% CI, 0.69-0.76), and 0.75 (95% CI, 0.72-0.78) for FRAX, SCORE, and OST, respectively, for identifying a femoral neck T score of –2.5 or lower.^22^ This study joins a body of literature on tools for predicting osteoporotic BMD and fracture risk among younger postmenopausal women aged 50 to 64 years.^17,20,21^ Despite its inclusion in the USPSTF osteoporosis screening guidelines, FRAX has been inferior in selecting postmenopausal women aged 50 to 64 years for osteoporosis screening with BMD testing.

### Limitations

Although the WHI provided a large sample, making the findings generalizable to a substantial number of younger postmenopausal women in the US, the study is not without limitation. WHI participants were healthier, had a higher educational level, and had higher socioeconomic status than the general population; thus, the results may not generalize to less healthy or more socially disadvantaged populations.

## Conclusions

In this cross-sectional study, we evaluated the performance of 3 USPSTF-recommended osteoporosis risk prediction tools—OSIRIS, ORAI, and OST—in younger postmenopausal women. We observed less than optimal performance (including discrimination), even after recalibration, in identifying osteoporosis as defined by a BMD T score of –2.5 or lower at any 1 of 3 skeletal sites. While the tools had good discrimination in identifying osteoporosis at the femoral neck, their fair to moderate discrimination in identifying osteoporosis at the hip or lumbar spine cast some doubt on the USPSTF recommendation to use these tools in clinical practice to identify younger postmenopausal women at risk for osteoporosis who may benefit from osteoporosis screening with BMD testing. Screening is essential to reduce the individual and societal burden of osteoporosis and related fractures, and this study showed a gap in identifying younger postmenopausal women with osteoporosis using common clinical risk factors. Future studies should examine whether alternative approaches, such as machine learning, can better identify younger women as appropriate candidates for osteoporosis screening.

## Notes

Supplement 1. **eTable.** Calculation of OST, ORAI, and OSIRIS Risk Scores

Supplement 2.
Data Sharing Statement
