# Supplementary material for: Identifying Younger Postmenopausal Women With Osteoporosis Using USPSTF-Recommended Osteoporosis Risk Assessment Tools
Source: JAMA Netw Open. 2025 Mar 18;8(3):e250626. doi: 10.1001/jamanetworkopen.2025.0626 (PMC11920839; doi:10.1001/jamanetworkopen.2025.0626)
Supplement: Supplement 1. — eTable. Calculation of OST, ORAI, and OSIRIS Risk Scores [file jamanetwopen-e250626-s001.pdf]

Supplemental Online Content

Zheng HW, Bui AAT, Ensrud KE, et al. Identifying younger postmenopausal women with osteoporosis using US Preventive Services Task Force–recommended osteoporosis risk assessment tools. *JAMA Netw Open*. 2025;8(3):e250626.
doi:10.1001/jamanetworkopen.2025.0626

eTable. Calculation of OST, ORAI, and OSIRIS Risk Scores

This supplemental material has been provided by the authors to give readers additional information about their work.

© 2025 Zheng HW et al. *JAMA Network Open*

eTable. Calculation of OST, ORAI, and OSIRIS Risk Scores

Tool	Feature		Score	Published Threshold
OSIRIS[11]	Age (years)		-years/5, rounded	<1
	Weight (kg)		kg/5, rounded	
	Current estrogen use		2	
	Prior low-impact fracture		-2	
ORAI[9]	Age (years)	45-54	0	>8
		55-64	5	
		65-74	9	
		75+	15	
	Weight (kg)	<60	9	
		60-69	3	
		70+	0	
	Not currently using estrogen		2	
OST[13]	Weight (kg)	(kg-years)/5, truncate to integer		<2
	Age (y)			

Raw score generated from sum of scores and rounded or truncated to nearest integer.

© 2025 Zheng HW et al. *JAMA Network Open*
