# Supplementary material for: Identifying Younger Postmenopausal Women With Osteoporosis Using USPSTF-Recommended Osteoporosis Risk Assessment Tools
Source: JAMA Netw Open. 2025 Mar 18;8(3):e250626. doi: 10.1001/jamanetworkopen.2025.0626 (PMC11920839; doi:10.1001/jamanetworkopen.2025.0626)
Supplement: Supplement 2. — Data Sharing Statement [file jamanetwopen-e250626-s002.pdf]

Data Sharing Statement

Zheng. Identifying Younger Postmenopausal Women With Osteoporosis Using US Preventive Services Task Force–Recommended Osteoporosis Risk Assessment Tools. *JAMA Netw Open*. Published March 12, 2025. doi:10.1001/jamanetworkopen.2025.0626

Data
Data available: Yes
Data types: Other (please specify)
Additional Information: Data Data available: Yes Data types: Deidentified participant data How to access data: National Heart, Lung, and Blood Institute at https://biolincc.nhlbi.nih.gov/home/ When available: With publication
How to access data: Data Data available: Yes Data types: Deidentified participant data How to access data: National Heart, Lung, and Blood Institute at https://biolincc.nhlbi.nih.gov/home/ When available: With publication
When available: With publication

Supporting Documents
Document types: None

Additional Information
Who can access the data: Data are publicly available; see above.
Types of analyses: For any purpose
Mechanisms of data availability: Without investigator support
